# A Causally Inspired Counterfactual Evaluation Framework for Wearable Assistive Robots

**DOI:** 10.3390/s26144646

**Published:** 2026-07-22

**Authors:** Wataru Fujita, Ryoma Tokunaga, Ai Higuchi, Tomohiro Shibata

**Affiliations:** 1 Department of Life Science and Systems Engineering, Graduate School of Life Science and Systems Engineering, Kyushu Institute of Technology, Kitakyushu 808-0196, Japan; tokunaga.ryoma1622@mail.kyutech.jp; 2 JTEKT Corporation, Kariya 448-8652, Japan; ai_higuchi@humanphysical.ai

**Keywords:** wearable assistive robots, time-series modeling, counterfactual evaluation, electromyography, human motion analysis, real-world data, assistive technology

## Abstract

Evaluating wearable assistive robots in real-world caregiving is challenging because temporally aligned and repeatable A/B comparisons are rarely available. Conventional evaluations assume that assist-on and assist-off trials are comparable in task content, posture, and movement timing. However, caregivers adjust their posture and timing across human–human interactions and task sequences. This study proposes a causally inspired diagnostic framework based on DBN/SCM-inspired time-series modeling and movement-fixed counterfactual estimation. We represented the multimodal observations using intervention, robot state, movement context, EMG, and context variables. Node-specific relationships were approximated using Attention-based Sparse Variational Gaussian Process regressors. We evaluated the framework at three levels of environmental complexity. These levels comprised controlled trunk flexion, partially controlled bed-to-wheelchair transfer, and real-world caregiving. The proposed framework is intended as a diagnostic counterfactual evaluation tool rather than as a method for strict causal identification. Across experiments, one-step EMG prediction accuracy alone was insufficient to identify intervention-sensitive models. In controlled validation, the selected movement-decoupled robot-only model reproduced an EMG-reducing response consistent with the controlled A/B reference. When fitted to the partially controlled transfer data, the selected structural specification identified an EMG-increasing response in supported contexts. In the real-world caregiving case study, the global assist-mediated response (AMR) was near zero despite a positive pooled A/B difference. However, the stratified analysis identified localized supported responses. The near-zero AMR indicates that the pooled difference was not reproduced through the modeled assist intervention–robot state–EMG pathway under fixed movement context. This result should not be interpreted as evidence of overall device ineffectiveness. These findings suggest that context-fixed counterfactual diagnosis can help interpret assistive responses under increasing environmental complexity.

## 1. Introduction

Wearable assistive robots have been developed to reduce physical workload during caregiving. They are particularly relevant to tasks involving repeated trunk flexion and patient handling. Low back pain and other musculoskeletal disorders remain major occupational health issues among caregivers, including in Japanese care settings [1,2]. Wearable assistive robotics is a promising approach to reducing this physical burden [3]. However, challenges remain in usability, adaptability, evaluation, and real-world deployment. While numerous studies have demonstrated the effectiveness of assistive devices in controlled laboratory environments, their evaluation in real-world settings remains a significant challenge. Conventional evaluation methods typically rely on A/B comparisons between assisted and unassisted conditions. These approaches assume that task execution is repeatable and temporally aligned, allowing direct comparison of physiological signals such as electromyography (EMG). However, in real-world caregiving environments, these assumptions rarely hold. Caregiving tasks are inherently heterogeneous. Transfers, repositioning, and physical support differ in duration, sequence, and intensity. Furthermore, assistive devices are often used intermittently, and assist-on and assist-off conditions are not synchronized across comparable movements. As a result, simple comparisons of averaged signals can lead to inconsistent or even misleading conclusions [4,5].

To address these limitations, recent studies have investigated data-driven approaches for estimating assistive effects from multimodal time-series data. These methods can capture complex relationships in multimodal sensor data. However, many rely on black box models and do not represent the physical interaction among assistive force, posture, and muscle activity. Moreover, many methods do not provide a principled estimate of counterfactual outcomes. Such estimates are needed to evaluate “what would have happened” under an alternative assist condition. The aim of this study is to introduce counterfactual reasoning as a diagnostic perspective for evaluating wearable assistive robots. The proposed framework does not claim strict causal identification. Instead, it applies movement-fixed counterfactual estimation (MF-CFE) under a fixed movement context. The purpose is to examine whether the learned pathway supports the observed assist-on/off difference. This pathway represents the model-implied response from the assist intervention to EMG through the robot state. Accordingly, the framework estimates the AMR through this learned pathway. It does not estimate effects mediated by changes in movement strategy.

In this study, we propose a causally inspired diagnostic framework for evaluating wearable assistive robots using heterogeneous multimodal time-series data. The framework does not assume that assist-on and assist-off observations are directly comparable. This is intended for real-world settings where movement and other contextual factors cannot be controlled. Instead, it examines whether the learned pathway supports the observed difference under a fixed movement context. This pathway links assist intervention to the EMG-based load response through the robot state. The multimodal representation includes assist intervention, robot state, movement context, EMG, and contextual variables. Attention-based Sparse Variational Gaussian Process regressors approximate the node-specific conditional relationships.

We examine the framework using a three-level protocol with increasing environmental complexity. The levels comprise controlled trunk flexion, partially controlled bed-to-wheelchair transfer, and real-world caregiving. Across these levels, the observed assist-on/off difference is interpreted differently depending on the degree of movement control and A/B comparability. The three-level protocol was designed to examine how the interpretation of a common diagnostic structure changes as experimental control decreases. Experiment 1 provided a controlled basis for selecting the primary structural specification. Experiment 2 retained a structured transfer task while allowing natural variation in movement execution, whereas Experiment 3 further introduced variability in task content, care recipients, assist timing, and human–human interaction. The same structural specification was retained across levels, while its parameters were estimated separately for each dataset. Thus, the protocol evaluated cross-level diagnostic consistency rather than the parameter-level transfer of a single fitted model.

**Figure 1:**
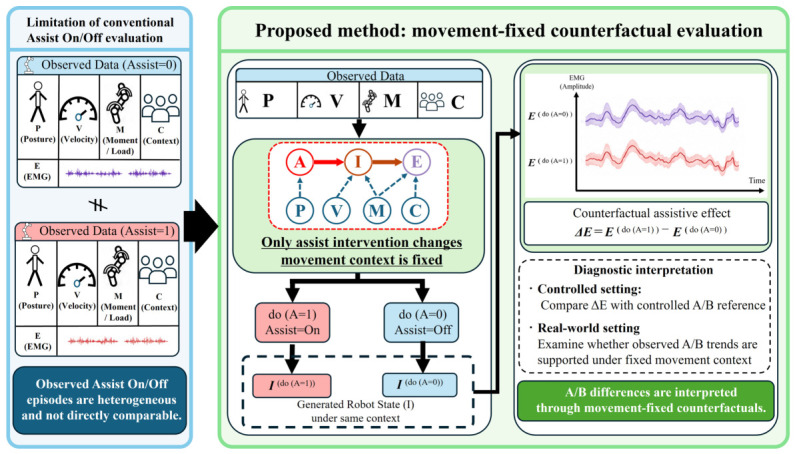
Conceptual motivation of the proposed movement-fixed counterfactual evaluation (MF-CFE). In real-world caregiving, a naive assist-off/on comparison can be misleading. Posture (*P*), velocity (*V*), the moment proxy (*M*), and task context (*C*) may differ across episodes. MF-CFE fixes the observed movement context and contrasts the two hypothetical intervention conditions, do(A=0) and do(A=1). The robot state trajectory (*I*) is generated through the learned assist intervention pathway, and the resulting EMG outcome (*E*) is predicted under each condition. The difference between the predicted EMG trajectories defines the assist-mediated response (AMR). The AMR is a model-implied diagnostic response under the same observed movement context. Accordingly, the AMR should be interpreted as a pathway-specific diagnostic response rather than as the total causal effect of device use.

Figure 1 illustrates the conceptual motivation of MF-CFE. In a naive A/B comparison, assist-on and assist-off episodes may differ in posture, velocity, load proxy, timing, and task context. MF-CFE therefore keeps the observed movement context fixed and changes only the hypothetical assist intervention. The model then predicts the robot state trajectory and EMG response under do(A=0) and do(A=1). The difference between the two predicted EMG trajectories is interpreted as the AMR. In this study, movement-fixed counterfactual evaluation refers to this rollout procedure, whereas context-fixed diagnosis refers to the stratified interpretation of these responses within comparable movement contexts.

A preliminary version of this framework was accepted for presentation at IEEE RO-MAN 2026 [6]. The present study extends the preliminary work in three respects. It reformulates the method as a movement-fixed diagnostic framework and separates the assist intervention from the robot state. It also introduces node-wise Attention-SVGP models and evaluates the framework across three levels of environmental complexity.

The main contributions of this study are summarized as follows:

- We propose a movement-fixed counterfactual evaluation (MF-CFE) framework for wearable assistive robots that does not require temporally aligned assist-on/off trials.
- We realize the framework using DBN/SCM-inspired node-wise modeling and Attention-SVGP regressors to represent robot state, movement context, and EMG relationships.
- We examine the framework across three levels, from controlled laboratory tasks to real-world caregiving. The interpretation of the observed assist-on/off difference depends on the experimental level.

## 2. Related Work

### 2.1. Evaluation Challenges in Wearable Assistive Robots

Wearable assist devices have been developed to reduce physical workload during caregiving and manual handling. These devices include rigid exoskeletons, soft exosuits, and powered transfer-support systems [7,8,9,10]. These devices are typically evaluated under controlled laboratory conditions. Common outcomes include EMG, kinematics, metabolic cost, subjective workload, and task-specific mechanical indices [11,12,13,14]. Controlled studies have reported reductions in muscle activity and perceived workload during lifting, sit-to-stand, and transfer-related tasks, demonstrating the potential of wearable assistive technologies [15].

However, conventional evaluations commonly rely on A/B or within-subject comparisons. These comparisons assume alignment in task phase, posture, timing, and external load. This assumption limits their applicability to real care environments, where task execution is rarely repeatable. Exoskeleton evaluation is also affected by task selection, sensor placement, outcome variability, and ecological validity [16]. In addition, device misalignment and user adaptation can also change trunk posture, movement trajectory, muscle activity, and timing. These changes may be greater when assistive forces conflict with the user’s intended motion [17].

Real-world nursing care adds variability that is absent from many laboratory tasks. Sources include spontaneous patient movement, human–human interaction, unpredictable timing, and cooperative load sharing [18]. Human–human physical interaction can alter movement dynamics and physical load because caregivers adapt their motion toward a shared task goal [19]. Such variability introduces temporal misalignment between assist and no-assist episodes, preventing straightforward comparison of averaged signals [20]. Moreover, confounding factors such as movement strategy and fatigue accumulation can substantially affect EMG outputs [16,21]. Recent evidence maps show substantial heterogeneity in occupational exoskeleton evaluation across healthcare, social care, and industry. Field evidence also remains limited compared with laboratory evidence [22].

### 2.2. Model-Based and Counterfactual Estimation

To address the limitations of direct A/B testing under heterogeneous movement conditions, model-based and causal-inference-inspired approaches have been introduced in related domains [23,24,25]. Counterfactual estimation provides a framework for asking what would have happened under an alternative intervention condition. Structural causal models formalize interventions and counterfactual outcomes [26], whereas Dynamic Bayesian Networks provide a probabilistic representation of temporal dependencies in time-series data [27]. These frameworks motivate structured modeling of assist state, robot state, movement context, and physiological outcomes in wearable assistive robot evaluation.

Although such approaches are not yet widespread in wearable assistive device evaluation, recent studies using deep causal models suggest their potential for HRI and biomedical applications [28]. Attention-based architectures have also been used to improve interpretability in time-series forecasting. For example, the Temporal Fusion Transformer combines recurrent layers, variable-selection networks, gating mechanisms, and interpretable self-attention for heterogeneous multi-horizon forecasting [29]. Recent Transformer-based approaches have further explored temporal causal discovery by extracting causal links and time lags from multivariate time-series forecasters while incorporating prior knowledge through attention masking [30]. These studies share our use of structured temporal dependencies and attention-based diagnostics. However, MF-CFE operates under a predefined intervention, robot state, movement context, and EMG structure. Thus, the framework is not a general black-box EMG predictor. It tests whether the learned A→I→E pathway supports an observed assist-on/off difference under fixed movement context.

### 2.3. Positioning of This Study

This study positions counterfactual time-series modeling as a diagnostic tool for evaluating wearable assistive robots under heterogeneous caregiving conditions. Rather than assuming that observed assist-on/off differences represent ground-truth assistive effects, the proposed framework examines whether such differences are supported by a learned intervention-sensitive robot state pathway under fixed movement context. The goal is not strict causal identification. Instead, the framework provides a structured diagnostic interpretation when temporally aligned A/B trials are unavailable.

## 3. Method

### 3.1. Overview

We formulated assistive effect evaluation as a movement-fixed counterfactual estimation problem from multimodal time-series data. The proposed framework estimates the model-implied change in EMG under alternative assist conditions while preserving the observed movement context. The model was inspired by Dynamic Bayesian Networks (DBNs) and Structural Causal Models (SCMs), which provide formal frameworks for representing temporal dependencies, interventions, and counterfactual outcomes [26,27]. However, the framework is not intended to establish strict causal identifiability. Instead, Attention-based Sparse Variational Gaussian Process (Attention-SVGP) regressors approximate the node-specific conditional relationships. Sparse inducing variables make the probabilistic regression scalable to large time-series datasets [31,32,33].

**Figure 2:**
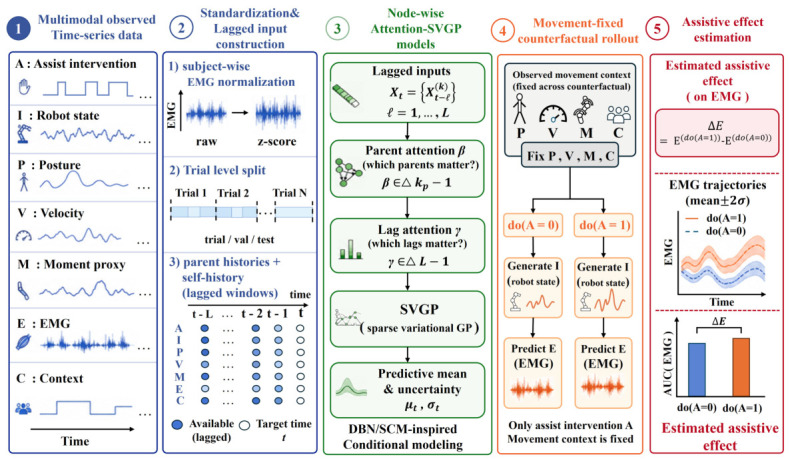
Overview of the proposed MF-CFE. Multimodal time-series data are standardized and converted into lagged parent histories for node-specific Attention-SVGP modeling. During counterfactual rollout, *A* is assigned to do(A=0) or do(A=1). The model generates *I* from the learned A→I relationship while fixing the observed *P*, *V*, and *M* trajectories. The assistive effect is quantified as the difference between the predicted EMG trajectories under the two intervention conditions.

The framework consists of four steps: (1) standardization of observed sensor data into intervention, robot state, movement, muscle-activity, and context variables; (2) construction of lagged parent histories and self-history features within each trial; (3) training of node-wise Attention-SVGP models; and (4) movement-fixed counterfactual rollout under do(A=0) and do(A=1). Figure 2 summarizes the overall workflow.

### 3.2. Variable Definition and Data Standardization

At each time step *t*, the standardized variable set was defined as

(1) $$X_{t}=\left\{A_{t},I_{t},P_{t},V_{t},M_{t},E_{t},C_{t}\right\},$$

where the variables are summarized in Table 1. Here, *t* denotes the time index, and $X_{t}$ denotes the complete standardized variable set at time *t*. The variables were designed to distinguish the explicit assist intervention, the robot state response, movement context, and EMG outcome.

A key design choice was the separation of the intervention variable $A_{t}$ and the robot state variable $I_{t}$. The assist intervention $A_{t}$ was derived from explicit assist-on/off labels, whereas $I_{t}$ was treated as a continuous robot state signal that could vary after intervention. This separation prevented assist-off periods with nonzero current values from being incorrectly treated as assist-on periods.

Posture $P_{t}$, velocity $V_{t}$, moment proxy $M_{t}$, and robot state variable $I_{t}$ were standardized using scalers fitted only on the training data. Let $E_{t}^{\mathrm{env}}$ denote the averaged EMG envelope before normalization. We transformed it as ${\tilde{E}}_{t}=\log\left(E_{t}^{\mathrm{env}}+\epsilon_{\mathrm{EMG}}\right)$, where $\epsilon_{\mathrm{EMG}}=10^{-8}$ was added only to avoid undefined values at zero. The logarithmic transformation compressed large amplitudes and reduced right-skewness in the EMG distribution. The transformed signal was then standardized within each subject using training-set statistics to reduce inter-subject amplitude differences. The context vector $C_{t}$ was constructed from one-hot representations of movement phase, subject identity, posture bin, and velocity bin. The posture and velocity bins were defined using quantiles estimated from the training set only. We split the data at the trial level. This procedure prevented temporally adjacent samples from the same trial from appearing in both sets.

### 3.3. DBN/SCM-Inspired Node Structure

**Figure 3:**
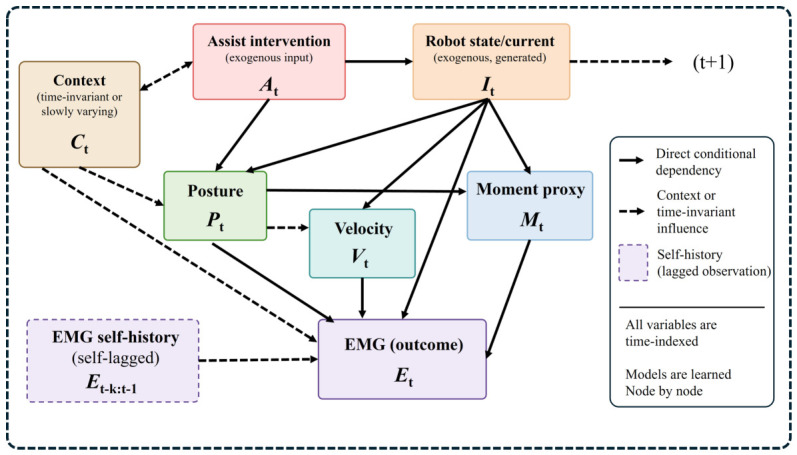
DBN/SCM-inspired node structure for counterfactual evaluation. The intervention node $A_{t}$ is treated as an intervention-like input, whereas the robot state node $I_{t}$ is modeled as an endogenous response. The remaining nodes represent movement context and EMG outcome variables defined in Section 3.2. Solid arrows denote direct conditional dependencies. Dashed arrows denote contextual or time-invariant influences, and the dashed box represents self-history features. The primary MF rollout fixes *P*, *V*, and *M*. It estimates the model-implied response through the A→I→E pathway rather than a fully identifiable causal effect. The moment proxy $M_{t}$ was treated as an observed and fixed input in the primary MF rollout rather than as a counterfactually generated node.

Figure 3 illustrates how the variables defined in Section 3.2 were organized into a DBN/SCM-inspired node structure. The structure follows the idea of representing temporally lagged dependencies among variables while distinguishing intervention-like inputs from endogenous responses [26,27]. However, the implementation used node-wise conditional regressors. It did not explicitly factorize a joint probability distribution.

Each node was modeled using a node-specific conditional function,

(2) $$Y_{t}=f_{Y}\left(C_{t},\mathrm{Pa}{\left(Y\right)}_{t-\ell },Y_{t-\ell_{s}}\right)+\epsilon_{Y},$$

where Y∈{I,P,V,E}, Pa(Y) denotes the parent set specified by the node structure in Figure 3, *ℓ* denotes external parent lags, $\ell_{s}$ denotes self-history lags, and $\epsilon_{Y}$ denotes observation noise.

The moment proxy $M_{t}$ was approximately calculated from trunk posture $P_{t}$, height, and body weight. This approximation follows a standard link-segment rationale in which anthropometric parameters and segment orientation are used to derive a posture-dependent external moment proxy [34]. Height and body weight were fixed subject-level inputs rather than time-varying nodes. The resulting $M_{t}$ trajectory was then treated as an observed biomechanical load-context proxy. During the primary MF rollout, the model fixed $P_{t}$, $V_{t}$, and $M_{t}$ to their observed trajectories. Thus, the rollout did not generate $M_{t}$ counterfactually.

This formulation is DBN-like because each node at time *t* is predicted from lagged parent variables and context variables. However, the model does not explicitly define a full joint distribution or estimate latent exogenous variables. Therefore, we refer to the model as a DBN/SCM-inspired counterfactual evaluation framework rather than a strict causal graphical model.

### 3.4. Lagged Input Construction

For each node *Y*, the input feature vector was constructed from the context vector, lagged parent histories, and, when enabled, self-history features

(3) $$X_{Y,t}=\left[C_{t},\mathrm{Pa}{\left(Y\right)}_{t-\ell },Y_{t-\ell_{s}}\right].$$

Lagged features were constructed within each trial so that histories did not cross trial boundaries. When self-history was disabled in an ablation setting, the self-history block was omitted. The EMG parent set was modified across model variants to examine the relative contribution of robot-related and movement-related pathways. The full model used {A,I,P,V,M} as EMG parents. The robot-only and mechanical models used {A,I} and {P,V,M}, respectively.

### 3.5. Attention-SVGP Regression Model

Each node-specific conditional relationship was approximated using an Attention-based Sparse Variational Gaussian Process (Attention-SVGP) regressor:

(4) $$y_{Y,t}=f_{Y}\left(X_{Y,t}\right)+\epsilon_{Y},\;\epsilon_{Y}∼N\left(0,\sigma_{Y}^{2}\right),$$

where $f_{Y}\left(\cdot \right)$ denotes a nonlinear probabilistic regression function. Here, $y_{Y,t}$ denotes the training target for node *Y* at time *t*, and $\sigma_{Y}^{2}$ denotes the node-specific observation-noise variance. The attention module summarized parent and lag histories using separate parent-level and lag-level weights. This design follows attention-based time-series forecasting models [29]. The SVGP module provided nonlinear regression with predictive uncertainty. Sparse variational approximations made this regression scalable [31,32,33]. Attention weights were used only as auxiliary indicators of predictive relevance and were not interpreted as causal effects [35,36]. Implementation details are provided in Appendix A.

### 3.6. Target Definition and Model Training

In the standard setting, the target variable for each node was its standardized current value:

(5) $$y_{Y,t}=Y_{t}.$$

For residual EMG models, the EMG target was defined as the change from a previous value:

(6) $$y_{E,t}^{\Delta }=E_{t}-E_{t-\ell }.$$

The residual target model yielded the closest agreement with the controlled A/B reference.

Each node-specific Attention-SVGP model was trained independently for Y∈{I,P,V,E}. Here, one-step prediction estimates $Y_{t}$ from observed lagged histories. All input histories come from the same trial. During this evaluation, the input histories were constructed from observed data and were not recursively replaced by model-generated outputs. Therefore, one-step accuracy measures short-horizon waveform fidelity. It is distinct from the counterfactual forward simulation described next.

We evaluated one-step prediction using Pearson’s correlation coefficient, RMSE, MAE, and mean predictive standard deviation. All metrics were calculated in standardized space.

### 3.7. Counterfactual Forward Simulation

After training, the learned node-specific models were used for counterfactual forward simulation. For each simulation window, the initial history was taken from the observed data, and the assist intervention was fixed to one of two hypothetical conditions:

(7) $$\mathrm{do}(A_{t}=0),$$

(8) $$\mathrm{do}(A_{t}=1).$$

Here, the intervention operator do(·) denotes the hypothetical assignment of the assist intervention variable independently of its observed value.

The main evaluation used a movement-fixed rollout (MF rollout). In each rollout branch, $A_{t}$ was assigned to 0 or 1. The learned A→I relationship then generated the robot state trajectory $I_{t}$. In contrast, the movement and biomechanical context variables $P_{t}$, $V_{t}$, and $M_{t}$ were fixed to their observed values throughout the simulation window. The model then predicted EMG under each intervention condition. Inputs comprised the generated robot state, fixed movement context, EMG self-history, and context variables.

This rollout addresses the following diagnostic question: Under the same observed movement context, how would EMG respond to an alternative assist condition and its associated robot state trajectory? Thus, the estimated effect represents the model-implied EMG change through the A→I→E pathway. It does not represent the total effect of assist use on movement strategy.

The frame-wise assistive effect on EMG was defined as

(9) $$\Delta E_{t}=E_{t}^{\mathrm{do}(A=1)}-E_{t}^{\mathrm{do}(A=0)}.$$

The AMR is the model-implied response through the learned A→I→E pathway under fixed movement context. It is not a fully identifiable causal effect.

Negative values of $\Delta E_{t}$ indicate reduced EMG activity under the assist-on condition, whereas positive values indicate increased EMG activity. The mean effect over a sequence was defined as

(10) $$\Delta E=\frac{1}{T}\sum_{t=1}^{T}\Delta E_{t}.$$

Here, *T* denotes the number of time frames in the evaluated sequence.

We refer to this model-implied EMG change as the assist-mediated response (AMR). Here, AMR denotes a diagnostic response transmitted through the learned assist intervention and robot state pathway under fixed movement context, rather than a fully identifiable causal effect. When both assist-on and assist-off observations were available, the observed A/B difference was computed as

(11) $$\Delta E_{\mathrm{AB}}=E\left[E|A=1\right]-E\left[E|A=0\right].$$

This value was used only as a level-dependent reference or descriptive statistic, not as a universal ground truth.

### 3.8. Ablation and Diagnostic Analyses

We trained structural variants to examine EMG prediction and counterfactual sensitivity. The variants changed the EMG parent set, self-history availability, target definition, or assist–robot state correspondence. The main variants are summarized in Table 2.

The full, robot-only, and mechanical models were used to compare the relative contributions of robot-related and movement-related pathways. No-self-history and residual target variants were introduced to test whether strong EMG self-history masked intervention-sensitive components of the EMG response. We introduced movement-decoupled variants because the assist intervention should not directly generate human movement trajectories. This restriction is important in practical caregiving tasks. In these models, the movement context variables *P*, *V*, and *M* were treated as fixed contextual variables during movement-fixed evaluation, while the robot-related pathway was represented through A→I→E.

Negative control analyses were performed by shuffling assist labels or disrupting the correspondence between assist state and robot state histories. The negative controls tested whether the estimated AMR depended on the learned A→I→E relationship. They also tested whether architecture, subject identity, phase distribution, or movement context alone could produce the response.

## 4. Experiment

### 4.1. Experimental Overview

**Figure 4:**
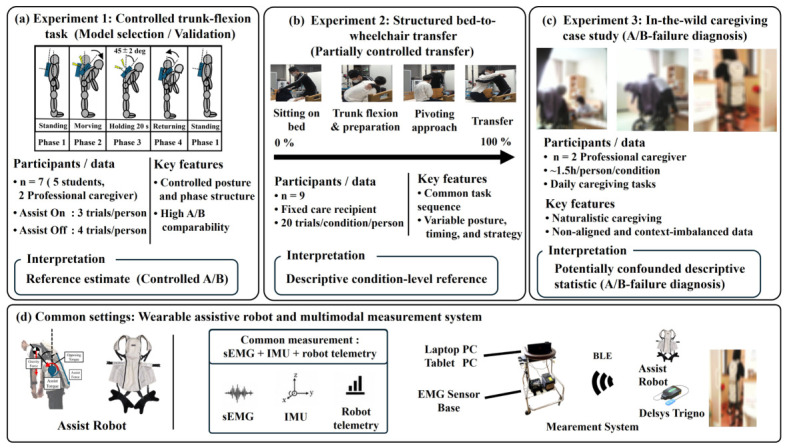
Overview of the three experimental levels and common measurement setup. The protocol consisted of (**a**) a controlled trunk flexion task for model selection and validation, (**b**) a partially controlled bed-to-wheelchair transfer task, (**c**) an in-the-wild caregiving case study for A/B-failure diagnosis, and (**d**) the common measurement setup used across the three levels, including the J-PAS fleairy wearable assistive robot, bilateral sEMG sensors, wearable IMUs, and robot telemetry. Each experimental panel summarizes the participant/data structure, task features, and interpretation of the observed assist-on/off comparison.

The proposed framework was evaluated using a three-level protocol with different degrees of movement control and A/B comparability. The protocol examined whether the learned A→I→E pathway supported each observed assist-on/off difference under fixed movement context. It did not treat every A/B difference as a ground-truth assistive effect. Table 3 summarizes the role of each level, and Figure 4 shows the common measurement platform and experimental protocol.

### 4.2. Measurement Setup and Signal Processing

The same wearable assistive robot and multimodal sensing platform were used across all experimental levels. The wearable assistive robot was J-PAS fleairy (JTEKT Corporation, Kariya, Japan), which provides extension assistance around the lumbar region during trunk flexion and caregiving-related postures. The measurement system included bilateral surface electromyography (sEMG) sensors, inertial measurement units (IMUs), and robot telemetry. Robot telemetry recorded assist activation and continuous device state signals. We mapped actuator current and internal device state to the intervention and robot state variables defined in Section 3.2.

sEMG was recorded bilaterally from the lumbar erector spinae muscles near the L4–L5 region using Delsys Avanti sensors (Delsys Inc., Natick, MA, USA). Raw EMG signals were acquired at 1926 Hz and resampled to 1000 Hz before signal preprocessing. After preprocessing and multimodal synchronization, the processed signals were downsampled to 20 Hz for model input, training, and evaluation. Electrode placement followed SENIAM recommendations [37]. We first applied a Hampel filter and a fourth-order 5–450 Hz band-pass filter. The signals were then full-wave rectified and smoothed using a 40-sample moving average. We averaged the bilateral EMG envelopes when both sensors were valid. When one sensor detached, we excluded that side and used the contralateral signal. The EMG amplitude was log-transformed and standardized separately within each subject, as described in Section 3.2. This preprocessing reduced amplitude skewness and inter-subject scale differences. It was not physiological normalization to maximum voluntary contraction because standardized MVC measurements were unavailable at every experimental level.

### 4.3. Experiment 1: Controlled Model Selection and Validation

Experiment 1 was conducted as a controlled trunk flexion task for model selection and validation. Participant/data details and trial counts are summarized in Figure 4a. The task consisted of repeated trunk flexion with controlled posture and phase structure, including forward bending, sustained flexion, and return to upright posture.

Because the task structure, target posture, and movement phases were controlled, the conventional assist-on/off EMG difference was treated as a controlled reference estimate. The hypothesis tested in this experiment was as follows:

**H1.**  *Under controlled laboratory conditions, the MF rollout reproduces the direction of the observed assist-on/off EMG difference. Its magnitude is also expected to be similar.*

Model variants were compared using AMRs, controlled reference discrepancy, negative control attenuation, and stratified directional consistency. The structural specification selected in Experiment 1 was retained in Experiments 2 and 3. Its parameters were estimated separately for each dataset.

### 4.4. Experiment 2: Partially Controlled Diagnostic Transfer

Experiment 2 evaluated the movement-decoupled robot-only model (MD-RO) in a partially controlled bed-to-wheelchair transfer task. The specification was fitted separately to the transfer dataset. Participant/data details and trial counts are summarized in Figure 4b. The task retained a common transfer sequence while allowing natural variability in posture, timing, and execution strategy.

Unlike Experiment 1, Experiment 2 did not prescribe the target trunk angle or movement duration. We therefore treated its observed A/B difference as a descriptive condition-level reference, not as ground truth. The hypothesis tested in this experiment was as follows:

**H2.**  *During partially controlled bed-to-wheelchair transfer, the framework estimates a dataset-specific AMR under variable movement execution. The response should depend on the selected structure and the validity of the robot state pathway.*

The primary analysis applied the model selected in Experiment 1. We also evaluated the MD-Full and PD-Full structures as sensitivity models. Negative controls tested whether the response depended on the learned correspondence between the assist intervention and robot state histories. Stratified analyses were performed across subject, movement phase, posture bin, velocity bin, and their combinations.

### 4.5. Experiment 3: Real Caregiving Case Study

Experiment 3 was a feasibility-oriented field case study. It evaluated the diagnostic value of the framework during practical caregiving. The field study used separate no-assist and assist-available recording days. Both sessions included the same predefined caregiving task categories. On the assist-available day, each caregiver could activate or deactivate the device according to the immediate situation. Thus, task categories were constrained at the session level. However, the order, duration, posture, assist timing, and local interaction context were not experimentally aligned. Participant/data details and recording duration are summarized in Figure 4c, and the task distribution is summarized in Table 4. The timing of assist activation under assist-on was left to the caregiver’s natural operation.

Unlike Experiments 1 and 2, Experiment 3 did not impose strict temporal or movement control. Task sequence, movement duration, posture, care recipient interaction, and assist timing varied naturally. Therefore, the observed A/B difference was interpreted as a potentially confounded descriptive statistic.

**H3.**  *In real-world caregiving, the framework tests whether the learned A→I→E pathway supports a large observed A/B difference. The test is performed under fixed movement context.*

The primary analysis examined whether the large observed A/B difference was reproduced by the MF rollout. A near-zero AMR indicated that the observed A/B difference was not reproduced through the modeled assist intervention–robot state–EMG pathway under the variables and assumptions used in this study.

### 4.6. Evaluation Metrics

The learned models were evaluated from three perspectives: predictive validity, counterfactual sensitivity, and diagnostic validity. This multi-perspective design was used because exoskeleton evaluation can be sensitive to measurement choices, task conditions, and outcome variability [16].

First, one-step EMG prediction performance was evaluated using Pearson’s correlation coefficient, RMSE, MAE, and mean predictive standard deviation in the standardized space.

Second, counterfactual sensitivity was evaluated using the AMR, quantified as the movement-fixed EMG change ΔE defined in Section 3.7. When both conditions were observed, we computed $\Delta E_{\mathrm{AB}}=E\left[E|A=1\right]-E\left[E|A=0\right]$. We summarized the discrepancy as $|\Delta E-\Delta E_{\mathrm{AB}}|$. The interpretation of $\Delta E_{\mathrm{AB}}$ followed the level-dependent definitions in Table 3.

Third, diagnostic validity was assessed using movement-fixed internal checks, negative control analyses, and stratified consistency. Because *P*, *V*, and *M* were fixed in the primary rollout, their counterfactual differences were expected to be zero. The generated robot state difference ΔI was used to verify whether the intervention propagated through the learned A→I pathway. Negative controls tested whether the AMR depended on the learned A→I→E pathway. They also assessed whether architecture or context distribution alone could produce the response.

For context-fixed diagnosis, each valid stratum contained at least 200 frames. Each assist-on/off condition also required at least 30 frames from two or more groups. A valid stratum was classified as supported when the observed $\Delta E_{\mathrm{AB}}$ and the model-implied ΔE had the same nonzero sign. In addition, the 95% bootstrap confidence intervals of both ΔE and ΔI were required to exclude zero. Confidence intervals were estimated using 2000 bootstrap resamples. A supported stratum was classified as strictly supported when the 95% bootstrap confidence interval of the observed $\Delta E_{\mathrm{AB}}$ also excluded zero. The sign of $\Delta E_{\mathrm{AB}}$ was used only to evaluate directional agreement with the AMR. In Experiment 1, it served as a controlled directional reference. In Experiments 2 and 3, it served only as a descriptive within-stratum reference. Therefore, the supported label does not treat the observed A/B difference as ground truth. It also does not provide evidence of causal validity. We defined the diagnostic logic before inspecting the stratified results. However, the numerical minimum-count thresholds were selected during the analysis and were not preregistered. Accordingly, these labels describe exploratory diagnostic consistency. They are not confirmatory validation criteria.

## 5. Results

### 5.1. Overview of Main Evaluation Strategy

The main cross-level results are summarized in Table 5. Experiment 1 served as the controlled model-selection stage, and the selected primary model was then applied to Experiments 2 and 3. Negative ΔE indicates an EMG-reducing AMR, whereas positive ΔE indicates an EMG-increasing AMR.

### 5.2. Experiment 1: Controlled Pathway Exploration and Model Selection

Experiment 1 served as the controlled structural selection stage. We retained the selected structural specification in Experiments 2 and 3 and estimated its parameters separately for each dataset. Because the trunk flexion task had a predefined phase structure and a controlled target posture, the observed assist-on/off difference was treated as a controlled reference. The observed A/B reference was $\Delta E_{\mathrm{AB}}=-0.110$, indicating lower mean EMG activity during assist-on.

Table 6 summarizes the comparison of structural variants. MD-RO produced an AMR of ΔE=−0.081. Its absolute discrepancy from the controlled A/B reference was 0.029, and the generated robot state difference was ΔI=+2.004. Thus, the intervention propagated through the learned A→I pathway while producing an EMG-reducing response.

The comparison showed that one-step prediction accuracy alone was insufficient for selecting a counterfactual evaluation model. The ablation robot-only and minimal models achieved higher one-step EMG prediction accuracy than MD-RO, but their AMRs were smaller or nearly absent. The residual target model showed the closest agreement with the controlled A/B reference. However, it had lower one-step prediction fidelity and did not provide the level-based EMG trajectory used in the primary MF rollout. We therefore retained it as a sensitivity model. Negative control models attenuated or eliminated the negative AMR, indicating that the response depended on the learned correspondence among assist state, robot state, and EMG.

We selected MD-RO as the primary structural specification. The decision considered controlled reference consistency, negative control attenuation, predictive performance, stratified directional consistency, and pathway interpretability.

**Figure 5:**
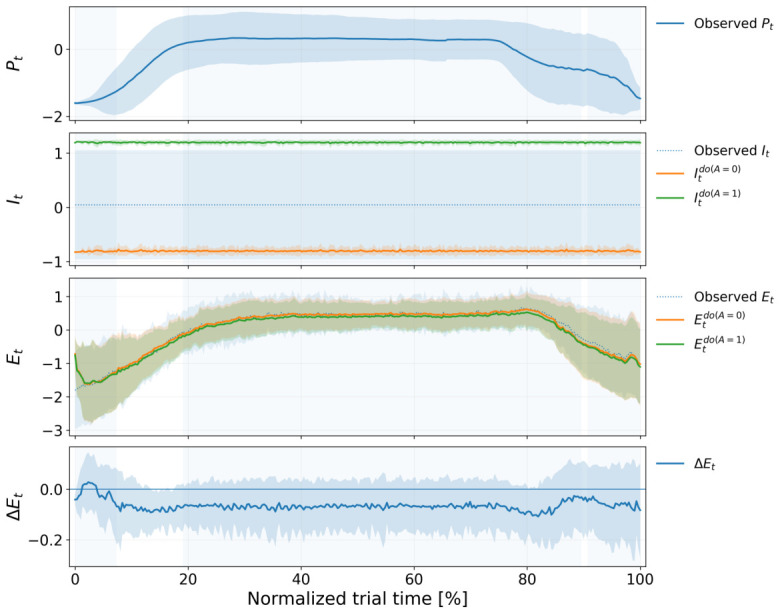
Group-level movement-fixed rollout of the selected primary model in Experiment 1. Controlled trunk flexion trials were time-normalized to 100% trial duration. Solid lines and shaded bands indicate the across-trial mean and ±1 standard deviation in standardized model space. The generated robot state trajectories separated clearly between do(A=0) and do(A=1). In contrast, the predicted EMG trajectories remained close. The frame-wise AMR $\Delta E_{t}$ was predominantly negative, indicating a small but consistent model-implied EMG reduction under assist-on.

Figure 5 shows the group-level MF rollout of the selected primary model in Experiment 1. The generated robot state trajectories separated clearly between do(A=0) and do(A=1), confirming propagation through the learned A→I pathway. The predicted EMG trajectories remained close in absolute magnitude. However, the frame-wise AMR was predominantly negative, indicating a small and consistent EMG reduction under assist on.

This model was not selected because it had the highest one-step prediction accuracy. Instead, MD-RO provided the most balanced evidence for an interpretable and intervention-sensitive pathway. This evidence was obtained under controlled movement conditions.

#### Context-Fixed Diagnosis in Experiment 1

After selecting MD-RO as the primary model, we examined whether the EMG-reducing response was supported within specific movement contexts. Table 7 summarizes the context-fixed diagnostic results. The supported ratios were high for single-context strata and became more selective when phase, posture, and velocity were combined. The selected structure did not impose a global negative response. Instead, it identified the movement contexts in which the learned A→I→E pathway supported the observed A/B difference.

Strictly supported contexts were mainly observed during sustained flexion and return phases, whereas early or low-load contexts showed weaker support. The selected structure localized the EMG-reducing AMR to mechanically relevant portions of the controlled task. It did not apply a uniform negative response across the trial.

### 5.3. Experiment 2: Partially Controlled Transfer and EMG-Increasing Response

Experiment 2 examined the MD-RO structural specification in a partially controlled bed-to-wheelchair transfer task. Its parameters were estimated from the transfer dataset. Experiment 2 provided less control over movement execution, load sharing, and human–human interaction than Experiment 1. We therefore treated its observed assist-on/off difference as a descriptive reference, not as ground truth.

The observed condition-level A/B difference was $\Delta E_{\mathrm{AB}}=+0.144$. The MF rollout using MD-RO estimated a positive AMR of ΔE=+0.072, with a generated robot state difference of ΔI=+0.370. Thus, in the partially controlled transfer task, the selected model detected an EMG-increasing response rather than an EMG-reducing response.

Table 8 summarizes the model comparison. MD-Full showed the closest agreement with the descriptive A/B difference, whereas PD-Full also produced a positive response. However, these models were treated as sensitivity models because primary model selection was anchored to the controlled Experiment 1. The negative controls strongly attenuated both ΔE and ΔI. This attenuation indicates that the positive response depended on the learned correspondence among the assist intervention, robot state, and EMG.

#### Context-Fixed Diagnosis in Experiment 2

Context-fixed diagnosis showed that the positive AMR was supported in a substantial subset of transfer contexts rather than uniformly across the entire task. As summarized in Table 9, support remained strong for single-context strata and became more selective when phase, posture, and velocity were combined. This indicates that the observed descriptive A/B difference was partially supported by the learned A→I→E pathway.

Representative supported contexts were concentrated in later transfer phases and in deeper posture or higher-velocity bins. Thus, the positive AMR was not uniform across the transfer task. It was concentrated in contexts with potentially greater stabilization or load-handling demands.

### 5.4. Experiment 3: Real Caregiving and Localized Supported Responses

Experiment 3 evaluated the framework using real-world caregiving data. Movement timing, task sequence, and human–human interaction were not experimentally aligned. The dataset comprised only two professional caregivers and provided no observable ground-truth counterfactual. Therefore, the results represent a feasibility-oriented field diagnosis, not population-level validation. This experiment was therefore treated as a real stress test for diagnosing whether observed assist-on/off differences were supported by the movement-fixed A→I→E pathway.

The observed assist-on/off difference was $\Delta E_{\mathrm{AB}}=+0.224$, indicating higher mean EMG activity during assist-on periods. However, the movement-fixed global AMR was close to zero: ΔE=−0.005, despite a positive generated robot state difference of ΔI=+0.314. Thus, the pooled assist-on/off difference was not reproduced under fixed movement context. Only the assist intervention and corresponding robot state trajectory differed between rollout branches. The near-zero global AMR indicates that the pooled A/B difference was not reproduced through the modeled A→I→E pathway under fixed *P*, *V*, and *M*. However, the near-zero AMR has two possible explanations. The modeled pathway may be weak, or the device effect may be mediated through movement or interaction variables outside that pathway. Therefore, it should not be interpreted as evidence of overall device ineffectiveness.

Table 10 summarizes the overall and subject-wise results. The subject-wise results were heterogeneous. Subject 0 had a large positive observed A/B difference but only a small positive AMR. Subject 1 had a near-zero observed difference and a small negative AMR. These results indicate that the pooled A/B difference in the real dataset should not be interpreted as a caregiver-independent global AMR.

#### Context-Fixed Diagnosis in Experiment 3

Without subject stratification, support was sparse. The supported ratios were 0.000 for phase and velocity, 0.200 for posture, and 0.108 for phase–posture–velocity strata. Without subject stratification, the most detailed movement context strata showed no strict support. These results indicate that the pooled A/B difference was not consistently supported after conditioning on movement context.

When subject identity was included in the context definition, supported contexts increased, but support appeared only when subject identity was combined with movement context variables.

Table 11 summarizes the subject-specific context-fixed diagnostic results. The subject-only stratum showed no support, whereas subject–phase, subject–phase–posture, and subject–phase–posture–velocity strata showed localized support. The real-world data did not support a global AMR shared across caregivers. However, the stratified analysis identified supported responses in specific caregiver–movement contexts.

Representative subject-specific strictly contexts are shown in Table 12. These examples identify caregiver–movement contexts in which the observed positive A/B difference is diagnostically supported. The pooled dataset did not support a global response.

## 6. Discussion

### 6.1. Why Movement-Fixed Counterfactual Evaluation Is Needed

This study proposed a movement-fixed counterfactual diagnostic framework for evaluating wearable assistive robots under heterogeneous caregiving conditions. The framework examines whether the learned A→I→E pathway supports an observed assist-on/off difference under fixed movement context. It does not estimate a universal assistive effect from pooled A/B averages.

Experiment 1 provided a controlled basis for selecting the primary structural specification. We retained this specification across Experiments 2 and 3 while estimating its parameters separately for each dataset. The cross-level evaluation therefore tested the diagnostic consistency of a common structural assumption, not the parameter-level transfer of a single fitted model.

The results also show how the interpretation of MF-CFE changes as experimental control decreases. In Experiment 2, the framework was tested under a structured transfer task with natural variation in movement execution. In Experiment 3, it was tested under broader heterogeneity in task content, task sequence, care recipients, assist timing, and human–human interaction. The attenuated global AMR in Experiment 3 suggests that the modeled variables were insufficient to represent a common response across this broader contextual range. The remaining variation may reflect unmodeled task details, caregiver strategy, care recipient behavior, fatigue, or interaction dynamics.

Thus, MF-CFE changes the evaluation question. It asks whether the observed difference is supported within comparable movement contexts, rather than whether assist-on changes the average EMG across all samples. This is particularly important in real caregiving data, where task content, posture, movement timing, and interaction with care recipients are not experimentally aligned. The controlled experiment supports the intervention sensitivity of the selected structural specification. However, it does not establish ground-truth validity after the specification is fitted to uncontrolled field data.

### 6.2. Task-Dependent AMRs and Trunk-Control Strategies

The sign reversal between Experiments 1 and 2 is informative. The selected structural specification estimated an EMG-reducing AMR in controlled trunk flexion. When fitted to the transfer data, it estimated an EMG-increasing AMR. This should not be interpreted as a contradiction. Rather, it suggests that the assist-mediated response depends on task demands and movement context.

This task-dependent sign change is biomechanically plausible. Different caregiving tasks require different trunk-control strategies, including load support, posture maintenance, balance correction, and interaction with the care recipient. Therefore, the robot may reduce lumbar extensor activity in one context while increasing stabilization demand, compensatory activation, or co-contraction-like control in another. The proposed framework is useful precisely because it does not force the assistive response to have a fixed direction. Instead, it evaluates whether the observed response is supported under fixed movement context. However, this interpretation is post hoc because the direction of the AMR was not prespecified and antagonist activity, muscle co-contraction, and caregiver–care recipient interaction force were not independently measured. It should therefore be regarded as a hypothesis for future biomechanical verification rather than as evidence of a specific physiological mechanism. The selected structural specification estimated an EMG-reducing AMR in controlled trunk flexion but an EMG-increasing AMR in the partially controlled transfer task. This contrast suggests that the modeled assist-mediated response depends on task demands and movement context rather than having a fixed direction. Previous studies have reported phase-specific increases in erector spinae activity during back-support exoskeleton use [38]. Exoskeleton-assisted tool handling has also increased estimated torso extensor forces and lumbar compression [39]. The distribution and magnitude of trunk muscle activity also vary across working tasks and exoskeleton mechanisms [13,40]. In addition, greater spinal-stability demands can promote trunk-muscle coactivation [41]. Accordingly, the positive AMR in Experiment 2 may reflect phase-specific loading, increased stabilization demand, or compensatory recruitment. However, this interpretation is post hoc because the AMR direction was not prespecified. We also did not independently measure antagonist activity, muscle co-contraction, or caregiver–care recipient interaction force.

The results also show that prediction accuracy alone is not sufficient for assistive effect diagnosis. Models with high one-step EMG prediction accuracy can rely on EMG self-history or movement context variables while showing little intervention sensitivity.

Residual target modeling increased agreement with the controlled A/B reference. However, it reduced level prediction fidelity and did not provide the EMG trajectory model used in the primary MF rollout. Therefore, model selection should balance prediction fidelity, controlled reference consistency, negative control attenuation, stratified support, and pathway interpretability.

### 6.3. Interpretation of Near-Zero AMR in Real Caregiving

The real caregiving case study demonstrated a failure mode of naive A/B comparison. The observed pooled A/B difference was positive, whereas the global movement-fixed AMR was near zero. The near-zero global AMR in Experiment 3 does not uniquely indicate that the device had little effect. It may arise either from a weak response through the modeled pathway or from an assistive response mediated through variables outside that pathway. The primary rollout did not estimate effects mediated through changes in posture, movement timing, stabilization strategy, or other behavioral adaptations. Fixing *P*, *V*, and *M* reduces imbalance in the measured movement context. However, it cannot remove differences in unobserved or coarsely represented context. Therefore, the field analysis shows that the modeled pathway did not reproduce the pooled A/B difference as a global AMR. It does not establish the absence of a total device effect. This does not imply that the device had no possible effect in any context. Rather, it indicates that the pooled A/B difference was not supported as a global caregiver-independent A→I→E response under fixed observed movement context.

This distinction is central to the diagnostic interpretation. In uncontrolled field data, assist-on and assist-off periods may differ in movement and interaction context. These differences can involve task composition, phase, caregiver behavior, care recipient interaction, and assist timing. A pooled A/B difference can therefore reflect contextual imbalance rather than a local assist-mediated response. By contrast, the context-fixed analysis identified localized subject-specific and movement-specific supported responses. These results suggest that real-world assistive-robot evaluation should focus on context-specific diagnostic interpretation rather than on pooled averages alone.

For practitioners, MF-CFE provides a staged interpretation of an observed assist-On/Off difference. The global AMR indicates whether the modeled pathway reproduces the pooled difference under fixed movement context. Stratified diagnosis then identifies the caregiver, task phase, posture-angle range, and movement condition associated with directional consistency. These context-specific results can support the redesign of assistive control rules. For example, an EMG-increasing, near-zero, or unsupported response in a particular phase or posture range may indicate that the assist timing, assistance magnitude, or control profile should be revised for that condition. In contrast, a stable EMG-reducing response can identify the conditions in which the current control strategy is most likely to be beneficial. Repeating the analysis after controller modification may further indicate whether the revised assistance improves previously unsupported contexts. In future applications, stratified response profiles may support pre-deployment assessment. They could provide users with a preliminary model-based estimate for each task, phase, and posture range. Such estimates should, however, be interpreted as preliminary diagnostic information rather than as guaranteed individual effects.

The sessions included matched caregiving task categories. However, self-selected assist timing may have concentrated assist-on periods in more demanding moments within each category. Fixing *P*, *V*, and *M* reduces imbalance in the measured movement variables. However, differences may remain in task stage, care recipient cooperation, interaction pattern, fatigue, or other unobserved context. Therefore, the field result is a pathway-specific diagnosis under the observed variables. It does not prove that all contextual imbalance was removed.

### 6.4. Limitations and Future Work

This study has several limitations. First, the proposed framework should not be interpreted as providing strict causal identifiability. The model was inspired by DBN and SCM concepts, but it did not estimate all exogenous variables, hidden confounders, or complete structural mechanisms. Therefore, the AMR is a model-implied diagnostic response under fixed observed movement context. It is not a fully identifiable causal effect.

Second, the MF rollout intentionally fixed posture, velocity, and moment proxy. This design isolates the local A→I→E pathway, but it does not estimate the total effect of assist use on movement strategy. In real use, assistive robots may change posture, timing, stabilization, or co-contraction. The primary rollout did not model these adaptation pathways as counterfactual outcomes. Future work should compare movement-fixed and movement-adaptive counterfactual models to separate local assist-mediated EMG responses from movement-strategy adaptation.

Third, the outcome variable was limited to processed lumbar erector spinae EMG. Lumbar erector spinae EMG is relevant to lumbar workload. However, assistive responses may also appear in other muscles, joint moments, perceived workload, balance, comfort, or task efficiency. Future analyses should incorporate additional physiological, biomechanical, and subjective outcomes.

Fourth, Experiment 3 was designed as a feasibility-oriented field case study rather than a population-level validation study. Experiment 3 involved only two professional caregivers, and the subject-specific findings should therefore not be generalized as caregiver-independent effect estimates. Larger real caregiving datasets are required to evaluate generalizability across users, care recipients, tasks, and device settings. The context vector $C_{t}$ did not fully represent several relevant factors. These factors included task labels, interaction forces, caregiver fatigue, movement strategy, and moment-to-moment changes in care recipient cooperation.

A further limitation is that care recipient-related factors were not explicitly observed or modeled. Care recipient characteristics may affect the caregiver’s posture, EMG activity, and robot use. Relevant characteristics include body size, residual motor ability, movement, cooperation, weight-bearing strategy, and interaction timing. These variables may therefore act as unobserved confounders, especially in field measurements involving uncontrolled human–human physical interaction. Future work should include care recipient-side measurements or annotations to improve the causal interpretability of real-world assistive effect diagnosis.

Finally, attention weights were used only as auxiliary indicators of predictive relevance. Attention weights were not the main evidence for intervention sensitivity. The evidence came from MF rollouts, context-fixed support, bootstrap stability, and negative control attenuation. Future work should examine personalized assistive control. These diagnostic outputs can directly identify redesign targets for assistive control. Supported EMG-reducing strata indicate task phases, posture-angle ranges, or movement conditions in which the current controller is likely to work as intended. In contrast, EMG-increasing, near-zero, or unsupported strata indicate conditions in which the activation timing, assistance magnitude, or cable-tension profile should be retuned.

## 7. Conclusions

This study proposed a causally inspired framework for diagnosing assistive responses in heterogeneous caregiving environments. The framework uses movement-fixed counterfactual time-series modeling. The framework tests whether the learned A→I→E pathway supports an observed assist-on/off difference under fixed movement context. It does not estimate a universally identifiable causal effect.

Across three experimental levels, the proposed framework revealed that assistive responses cannot be adequately interpreted from prediction accuracy or pooled A/B comparisons alone. In the controlled trunk flexion experiment, the selected MD-RO reproduced an EMG-reducing AMR and provided a controlled anchor for model selection. When fitted to the partially controlled transfer data, the selected structural specification identified an EMG-increasing response. This result shows that the framework did not impose a fixed bias toward EMG reduction. In a real-world experiment, the learned pathway did not support the large pooled A/B difference as a global AMR. However, the stratified analysis identified supported responses in specific caregiver–movement contexts. Accordingly, a near-zero AMR indicates weak support for the modeled A→I→E component under fixed movement context. It does not establish the absence of a total device effect.

Real-world evaluation should not focus only on whether assist-on changes the average EMG; it should also identify the movement contexts in which the learned pathway supports the observed difference. The proposed context-fixed counterfactual diagnosis may therefore help distinguish locally supported responses from potentially confounded condition-level differences and identify contexts that require further controlled or biomechanical evaluation.

Future work should extend the framework to larger real datasets, richer context variables, additional biomechanical and subjective outcomes, and movement-adaptive counterfactual models. These extensions may improve the reliability of wearable robot evaluation. They may also support personalized control that adapts assistance to user-specific and task-specific response patterns.

## Figures and Tables

**Table 1:** Variables used in the proposed framework.

Symbol	Variable	Definition and Role
$A_{t}$	Assist intervention	Binary assist-on/off intervention label
$I_{t}$	Robot state	Continuous robot telemetry or current-related state
$P_{t}$	Posture	Trunk posture angle
$V_{t}$	Velocity	Trunk angular velocity computed within each trial
$M_{t}$	Moment proxy	Approximate biomechanical load-context variable
$E_{t}$	EMG	Lumbar erector spinae EMG envelope
$C_{t}$	Context	Phase, subject, posture bin, and velocity bin information

**Table 2:** Structural model variants used for diagnostic evaluation.

Model Variant	EMG Parent Set	Self-History/Target	Diagnostic Role
Full	{A,I,P,V,M}	EMG level with self-history	Reference model including robot and movement context parents
Robot-only (RO)	{A,I}	EMG level with self-history	Isolates the assist intervention–robot state pathway for EMG prediction
Mechanical	{P,V,M}	EMG level with self-history	Evaluates movement- and mechanics-related EMG prediction
No E-self	Model-specific parent set	EMG self-history removed	Tests whether EMG self-history masks intervention sensitivity
Residual target	Model-specific parent set	Residual EMG target	Tests whether predicting EMG change increases intervention sensitivity
Movement-decoupled robot-only (MD-RO)	{A,I}	EMG level with self-history	Primary model for the movement-fixed modeled A→I→E pathway
Movement-decoupled full (MD-Full)	{A,I,P,V,M}	EMG level with self-history	Sensitivity model including movement context parents for EMG
P-decoupled full (PD-Full)	{A,I,P,V,M}	EMG level with self-history	Intermediate sensitivity model with posture generation decoupled
NC shuffle-A	Same as corresponding original model	Same as corresponding original model	Negative control with shuffled assist labels
NC shuffle-AI	Same as corresponding original model	Same as corresponding original model	Negative control disrupting assist–robot state correspondence

Note: *A*, assist intervention; *I*, robot state; *P*, posture; *V*, velocity; *M*, moment proxy; EMG, electromyography; RO, robot-only; MD, movement-decoupled; PD, posture-decoupled; NC, negative control.

**Table 3:** Experimental levels and interpretation of A/B comparison.

Level	Experiment	Role	Interpretation of A/B Comparison
Level 1	Controlled trunk flexion task	Controlled model selection and validation	Reference estimate
Level 2	Partially controlled bed-to-wheelchair transfer	Partially controlled diagnostic transfer	Descriptive condition-level reference
Level 3	Real-world caregiving case study	A/B-failure diagnosis	Potentially confounded descriptive statistic

**Table 4:** Task distribution in the real caregiving case study.

Task	Assist Off	Assist On	Mean Duration [s]
Bathing	4	3	1309.0
Transfer	4	3	231.0
Dressing	8	9	358.8
Toilet	3	4	236.5
Other	6	11	103.2

**Table 5:** Cross-level summary of observed A/B differences and AMRs.

Experiment	Model	Observed $\Delta E_{\mathrm{AB}}$	AMR ΔE	Interpretation
EXP 1	MD-RO	−0.110	−0.081	Controlled reference compatible
EXP 2	MD-RO	+0.144	+0.072	Same direction as descriptive A/B
EXP 2	MD-Full	+0.144	+0.105	Closest descriptive agreement
EXP 3	MD-RO	+0.224	−0.005	A/B not reproduced
EXP 3	MD-Full	+0.224	0.000	Near-zero AMR

**Table 6:** Experiment 1 controlled model selection. The observed A/B reference was $\Delta E_{\mathrm{AB}}=-0.110$.

Model	*E* Corr.	*E* RMSE	AMR ΔE	Error	ΔI	Role
MD-RO	0.990	0.143	−0.081	0.029	2.004	Primary
Ablation RO	0.998	0.071	−0.053	0.057	2.027	Predictive baseline
Minimal	0.998	0.068	−0.003	0.106	2.026	Prediction-only baseline
MD-Full	0.990	0.140	+0.000	0.110	2.023	Sensitivity
PD-Full	0.991	0.138	−0.005	0.105	2.015	Sensitivity
Residual target	0.947	0.353	−0.106	0.004	–	Diagnostic sensitivity
NC-A	0.990	0.141	+0.000	–	–	Negative control
NC-AI	0.990	0.143	+0.018	–	–	Negative control

**Table 7:** Context-fixed diagnostic summary in Experiment 1.

Context Stratum	Supported	Strict	Mean ΔE
Phase	0.750	0.250	−0.081
Posture	1.000	0.000	−0.080
Velocity	1.000	0.200	−0.081
Phase-posture	0.875	0.188	−0.109
Phase-velocity	0.800	0.100	−0.071
Posture-velocity	0.840	0.040	−0.078
Phase-posture-velocity	0.475	0.038	−0.096

**Table 8:** Experiment 2 model comparison. The observed condition-level A/B difference was $\Delta E_{\mathrm{AB}}=+0.144$.

Model	$r_{E}$	RMSE_*E*_	AMR ΔE	Error	ΔI
MD-RO	0.970	0.243	+0.072	0.072	+0.370
MD-Full	0.970	0.244	+0.105	0.039	+0.481
PD-Full	0.970	0.240	+0.092	0.052	+0.334
NC-A	0.969	0.245	−0.008	0.152	+0.007
NC-AI	0.969	0.244	+0.022	0.122	−0.011

**Table 9:** Context-fixed diagnostic summary in Experiment 2.

Context Stratum	Supported Ratio	Strict Ratio
Phase	0.750	0.500
Posture	0.600	0.600
Velocity	1.000	1.000
Phase-posture	0.556	0.444
Phase-velocity	0.600	0.450
Posture-velocity	0.600	0.560
Phase-posture-velocity	0.477	0.326

**Table 10:** Overall and subject-wise results in Experiment 3.

Level	Samples	$n_{A=0}$	$n_{A=1}$	$\Delta E_{\mathrm{AB}}$	ΔE	ΔI
Overall	405,856	210,558	195,298	+0.224	−0.005	+0.314
Subject 0	224,506	112,239	112,267	+0.391	+0.022	+0.230
Subject 1	181,350	98,319	83,031	+0.029	−0.039	+0.417

**Table 11:** Subject-specific context-fixed diagnostic summary in Experiment 3.

Context Stratum	Supported Ratio	Strict Ratio
Subject	0.000	0.000
Subject-phase	0.125	0.125
Subject-phase-posture	0.192	0.058
Subject-phase-posture-velocity	0.226	0.049

**Table 12:** Representative subject-specific strictly supported contexts in Experiment 3.

Context	$\Delta E_{\mathrm{AB}}$	ΔE	ΔI
Subject 0 phase 3 posture 1	+0.332	+0.101	+0.080
Subject 0 phase 2 posture 1 velocity 3	+0.541	+0.091	+0.025
Subject 0 phase 2 posture 3 velocity 3	+0.586	+0.074	+0.358
Subject 0 phase 3	+0.304	+0.061	+0.071

## Data Availability

The data are not publicly available due to privacy and ethical restrictions involving human participants in caregiving environments.

## Abbreviations

The following abbreviations are used in this manuscript:

A/B	Assist-On/Off comparison
AMR	Model-implied assist-mediated response
DBN	Dynamic Bayesian Network
EMG	Electromyography
GP	Gaussian Process
HRI	Human–Robot Interaction
IMU	Inertial Measurement Unit
MAE	Mean Absolute Error
MD-Full	Movement-decoupled full model
MD-RO	Movement-decoupled robot-only model
MF-CFE	Movement-fixed counterfactual evaluation
MF rollout	Movement-fixed rollout
NC	Negative control
PD-Full	P-decoupled full model
RMSE	Root Mean Square Error
SCM	Structural Causal Model
sEMG	Surface electromyography
SVGP	Sparse Variational Gaussian Process

## Appendix A. Attention-SVGP Implementation Details

The Attention-SVGP model used parent-level attention β and lag-level attention γ to summarize lagged parent histories before applying an SVGP regression module. The SVGP component used inducing-point sparse variational Gaussian process regression with a Gaussian likelihood and a variational evidence lower bound objective. Attention weights were used only as auxiliary indicators of predictive relevance and were not interpreted as causal effects.
